# The Role of Social Appearance Comparison in Body Dissatisfaction of Adolescent Boys and Girls

**DOI:** 10.5964/ejop.6443

**Published:** 2023-08-31

**Authors:** Lucia Barbierik, Maria Bacikova-Sleskova, Veronika Petrovova

**Affiliations:** 1 Department of Educational Psychology and Health Psychology, Pavol Jozef Šafárik University in Košice, Košice, Slovak Republic; University of Palermo (UNIPA), Palermo, Italy

**Keywords:** internalization of societal ideals of appearance, social appearance comparison, body dissatisfaction, adolescents

## Abstract

The main aim of the present study is to investigate the indirect effect of the association between thin-ideal internalisation (1), muscular-ideal internalization (2) and body dissatisfaction (BD) through the general social appearance comparison separately among boys and girls. 154 adolescents (mean age 18.2 years, SD = 0.73; 56.5% girls) provided information on the explored variables. Two hierarchical regression models were carried out for boys and girls separately. The general appearance comparison works as an important explanatory mechanism in the relationship between thin-ideal internalization and BD among girls as well as between muscular-ideal internalization and BD among both boys and girls. The more individuals internalize the societal ideals of appearance, the more they compare their physical appearance to others and thus the greater BD they perceive. The current results contribute to previous research findings by indicating the social appearance comparison as a risk factor which enhances BD among both boys and girls in late adolescence. The findings may facilitate identifying individuals who are vulnerable to body dissatisfaction earlier, before more serious eating problems occur.

Young people are at particular risk of social comparisons of appearance in both the real and online world (Ho et al., 2016; Ricciardelli & Yager, 2016). Individuals tend to compare themselves with others in order to gain information of “how they fit into the world” when objective standards are missing or when they are unsure of their own self-evaluation (Fitzsimmons-Craft et al., 2016). A negative perception of self when comparing to others can have an adverse effect on mental health, well-being and physical self-esteem. Furthermore, this can lead to growing body dissatisfaction and the consequences of “too healthy" a lifestyle (restrictive eating, excessive exercising or other rigid ways of weight control) (Rancourt et al., 2016; Thompson & Stice, 2001; Vartanian & Dey, 2013). The media is another negative influence with the presentation of unattainable and perfect male or female figures (Ricciardelli & Yager, 2016). Those figures are typically viewed as the representation of health, success and social desirability (Thompson et al., 2004; Van Vonderen & Kinnally, 2012). However, it is not the awareness of standards of appearance but its internalization which has been found to be responsible for increased body dissatisfaction (Cafri et al., 2005; Vartanian & Dey, 2013). The current study discusses the role of both social appearance comparisons and internalization of societal ideals of appearance in the body dissatisfaction of boys and girls in late adolescence. Furthermore, it looks at gender differences in these concepts as well as the role of gender in the examined associations.

## Body Dissatisfaction

Body dissatisfaction is usually understood as the negative thoughts and feelings towards one´s own body or the negative evaluation of body shape, weight, body parts or global appearance (Grogan, 2016). It is a complex phenomenon explained by various socio-demographic, biological or psychological factors. The strongest predictor of body dissatisfaction is being a women and body weight or body mass index (Esnaola et al., 2010; Ricciardelli & Yager, 2016) besides other physical and biological factors such as early maturation in puberty or genetic influences (Ricciardelli & Yager, 2016). From individual psychological factors, personality traits were found to be associated with higher body dissatisfaction (Allen & Walter, 2016) as well as depressive symptoms, lower self-esteem (Wang et al., 2019) and greater internalization of societal ideals (Ricciardelli & Yager, 2016). Moreover, environmental factors such as social appearance comparison (Carlson Jones, 2004), lower parent-child connectedness, more frequent weight teasing and peer dieting predicted increased body dissatisfaction (Wang et al., 2019). Previous research has provided substantial evidence of the relationship between attitudes to one’s own body, psychosocial functioning and well-being (Carraça et al., 2011). Body dissatisfaction has been found to be associated with poor quality of life (Horacek, 2013). Moreover, it is considered to be an important predictor of psychopathologies in adolescence such as unhealthy weight control strategies (Keery et al., 2004), mood disorders (Barnes et al., 2020; Sharpe et al., 2018) and abnormal eating behaviour (Allen et al., 2008) and even eating disorders (Shagar et al., 2017; Tod & Edwards, 2015).

Dissatisfaction with physical appearance can occur among both men and women at different ages (Quittkat et al., 2019). While early adolescence is a critical period (Wang et al., 2019), it is most prevalent during adolescence and adulthood (Ricciardelli & Yager, 2016). Adolescents are used to assessing their bodies with respect to sociocultural attractivity norms (Ricciardelli & Yager, 2016). In adolescence, a girl's identity is influenced by societal demands for thinness and physical beauty while physical height, strength, muscularity, attractiveness as well as the functional aspect of body are very important factors of social competence for boys (Giles, 2003; Ricciardelli & Yager, 2016; Vágnerová, 2000). Adolescents often use comparing appearances with their peers or internalizing an ideal body appearance to attain social acceptance and prestige (Vágnerová, 2000). The social appearance comparison process and internalization of societal ideals of appearance have been considered key psychological factors in explaining body dissatisfaction of adolescents (Carlson Jones, 2004; Paterna et al., 2021; Ricciardelli & Yager, 2016).

## Social Appearance Comparison and Internalization of Societal Ideals of Appearance

The Tripartite Influence Model of Body Image and Eating Disturbance (Thompson et al., 1999) explains body dissatisfaction and eating disorders through the mechanism of social pressures (peers, family and media). These lead individuals to internalize socially and culturally appreciated appearance ideals. The internalization of sociocultural ideals is usually defined as the “adoption of societal appearance ideals as a personal goal and standard” (Carlson Jones, 2004, p. 823). In other words, it refers to the incorporation of specific external values in guiding one’s own principles (Thompson & Stice, 2001). The extent of it can vary from beliefs in a set of ideas about societal norms of size and appearance to behavioural changes in an attempt to approximate these sociocultural standards of appearance (Thompson & Stice, 2001). Societal ideals of appearance are often operationalized as those which are conveyed by the media (Thompson et al., 2004). These take the form of usually unattainable, perfect figures of male or female bodies in television programmes, magazines, commercials and these days, social networks. These perfect figures, their body weight, shape and beauty are typically viewed as the representation of health, success and social desirability. In contrast, overweight figures are frequently ridiculed in the media (Van Vonderen & Kinnally, 2012). An awareness of standards of appearance itself at a conscious level cannot lead to body dissatisfaction. However, its internalization as a more latent self-schema explains how sociocultural pressures affect one’s body image (Cafri et al., 2005). Consequently, those who fail to achieve these societal ideals will experience negative feelings about their bodies (Vartanian & Dey, 2013).

The Tripartite Influence Model (Thompson et al., 1999) also suggests a role for social appearance comparison in body image disturbance. It refers to an evaluative process involving both seeking information and making judgments about one’s own physical attributes compared with those of others. According to Festinger’s social comparison theory (1954, as cited in Fitzsimmons-Craft et al., 2016), individuals compare themselves with others in order to understand how they fit into the world when objective standards are not available. Such comparisons are more frequent among individuals with lower self-esteem and those more uncertain of their own self-evaluations (Carlson Jones, 2004). They can either do it passively (somewhat automatically and subconsciously) or actively (goal-directed behaviour) by selecting a suitable comparison ‘target’ (Blowers et al., 2003). Individuals tend to either compare themselves to superior individuals (upward comparisons) or to less attractive ones (downward comparisons), resulting in a different effect regarding self-evaluation, body dissatisfaction and eating disorders (Rancourt et al., 2016). More often than not however, appearance comparisons tend to be of upward nature (Ko et al., 2019). In addition, the general chronic tendency to make appearance-based comparisons (a trait level approach), regardless of the direction, is associated with negative outcomes (Vartanian & Dey, 2013). Thus, this internalization and comparison lead to body dissatisfaction, commonly defined as a risk factor for unhealthy weight control strategies and disordered eating (Thompson & Stice, 2001).

## Gender Specificity

The gender specific approach to the topic of body dissatisfaction has been criticized by some authors. Indeed, they have argued that this is no longer women’s gender issue and that body image concerns have changed in recent decades (Carlson Jones, 2004; Tantleff-Dunn et al., 2011). The large number of men undergoing cosmetic surgery over the past few years points to the importance of exploring the body dissatisfaction of men as well as women (Matera et al., 2018). Attitudes and behaviours related to body image and appearance improvement are common for men too, even though the idealized physical appearance may differ across gender (Murray et al., 2017).

With regards to the internalisation of societal ideals of appearance, current research differentiates the slim or thin body ideal, when explaining women’s body dissatisfaction and the muscular or mesomorphic body ideal, typical for men’s body dissatisfaction (Karazsia et al., 2017). Even though the main research interest is devoted to the appearance ideals and body dissatisfaction of women, there is also evidence that internalized appearance ideals significantly contribute to the body dissatisfaction of men (Carlson Jones, 2004). While the thinness-oriented body dissatisfaction has declined over the years among women, muscularity-oriented dissatisfaction has remained stable in men (Karazsia et al., 2017). In men, there are two dimensions of body ideal internalization, thinness and muscularity, which are associated with body dissatisfaction and eating concerns. It seems that the thin-ideal internalization is more strongly associated with body dissatisfaction and eating disturbances, when compared to muscular ideal internalization, also among men (Paterna et al., 2021; Schaefer et al., 2021). It could be explained by the fact that muscular-body ideal is easier to attain in comparison to the thin-body ideal or general attractiveness (Paterna et al., 2021). Regarding the thin-ideal internalization among men, disordered eating behaviours may include dieting to lose weight as well as making muscularity more visible (Klimek et al., 2018). Moreover, some authors emphasize that the ideal body conveyed by the media focuses on exercise, muscularity and athleticism for both men and women (Tylka, 2011). In addition, it seems that muscularity and thinness or leanness are both desired characteristics for men and women (Paterna et al., 2021; Watson & Murnen, 2019). That is why the current study includes both the muscularity and thinness internalization components.

Independently of the targets of comparison (upward or downward comparisons), social appearance comparison has previously been found to be positively associated with body dissatisfaction among early and middle adolescent boys and girls (Carlson Jones, 2004) as well as undergraduate men and women (Ko et al., 2019). The associations were stronger for women and inversely related to age (Carlson Jones, 2004; Myers, & Crowther, 2009). However, it has also been found that the peer appearance context did not play a significant role in the more complex mediational models explaining body dissatisfaction among early and middle adolescent boys (Carlson Jones, 2004). Women are embedded in the appearance culture from an early age (Thompson et al., 1999) whereas men move into the cultural glorification of muscularity associated with manhood during the secondary school and university years (Carlson Jones, 2004). Thus, the empirical part of the current study addresses boys and girls in late adolescence.

## Links Between Internalization, Social Comparison and Body Dissatisfaction

The role of internalization of appearance ideals and social comparison in explaining body dissatisfaction is still being debated with research bringing mixed results (Vartanian & Dey, 2013).

Social comparison could work as a moderator of the relationship between internalisation and body dissatisfaction. It has been found that superior or upward comparisons interacting with a high level of internalization of the thin ideal lead to higher body dissatisfaction among women, while non-superior or downward comparisons lead to lower body dissatisfaction in women, independent of the level of internalization (Cattarin et al., 2000). However, general social comparison did not moderate the association between internalisation of the thin ideal and body dissatisfaction among primary school aged girls (Blowers et al., 2003).

Another possible explanatory mechanism of body dissatisfaction proposes internalisation as a mediator. Individuals who are highly attentive to social comparison may come to see the advantages associated with certain appearance features and adopt the ideal images as their own personal goal (Carlson Jones, 2004). Thin-ideal internalisation was found to mediate the relationship between upward comparisons and body dissatisfaction among undergraduate women (Vartanian & Dey, 2013). Those findings suggest that repeatedly comparing one’s appearance to superior ones may facilitate thin-ideal internalization, which in turn increases body dissatisfaction. However, in this alternative, other factors such as lower self-concept clarity played a significant role as well.

A different view suggests social comparison as a mediator in the relationship between internalization of appearance ideals and body dissatisfaction. The internalization of the appearance ideal may stimulate physical appearance comparisons to let individuals see how close they are to their ideal by comparing themselves to others. Adverse findings can in turn increase body dissatisfaction. Body comparison at the trait level was found to be a mediator between thin ideal internalization and body dissatisfaction among undergraduate women (Fitzsimmons-Craft et al., 2016), pre-adolescent (Blowers et al., 2003) and middle adolescent girls (Durkin et al., 2007).

The current study follows the last paradigm, explaining the mechanism as to how the internalization of appearance ideals leads to body dissatisfaction through the social appearance comparison. In other words, social comparison facilitated by internalization of appearance ideals enables individuals to come to know how good/bad they are at approximating such an ideal in comparison to others. To the best of our knowledge, this has only been supported with respect to women (Blowers et al., 2003; Durkin et al., 2007; Fitzsimmons-Craft et al., 2016). In addition, previous research has also demonstrated the importance of general social appearance comparison at the trait level in the context of body dissatisfaction (Tiggemann & McGill, 2004) and the general appearance comparison tendency and its negative impact on eating disturbance (Vartanian & Dey, 2013). Therefore, this study is focused on general appearance comparisons as well.

## The Aim of the Current Study

Despite clear evidence of the adverse effects of thin-ideal internalization and appearance-related social comparison on body dissatisfaction (Fitzsimmons-Craft et al., 2012), there is a need to examine the connection between these two risk factors and further explain the role that they have in explaining body dissatisfaction. Even though studies regarding body dissatisfaction, appearance comparison and ideal body internalization are more frequent among women and associations tend to be stronger among women in comparison to men (Carlson Jones, 2004; Lawler & Nixon, 2011), it is important to focus on men as well (Matera et al., 2018; Tylka, 2011). The ideal body internalization and social comparison of physical appearance may represent key psychological factors that facilitate body dissatisfaction among both adolescent boys and girls (Lawler & Nixon, 2011) as well as young adult men and women (Ko et al., 2019; Matera et al., 2018; Strubel & Petrie, 2017). The current study expands previous findings by focusing on boys in late adolescence in order to determine whether social comparison arises as a significant factor in the development of body dissatisfaction at this age. The psychological as well as physical development of boys tends to be slower and the importance of body appearance may arise later than it does among girls (Carlson Jones, 2004; Ricciardelli & Yager, 2016). When exploring both genders, it is important to address both thin-ideal and muscular/athletic-ideal internalisation (Tylka, 2011). Thus, the aim of present study is to investigate the indirect effect of the association between thin-ideal internalisation, muscular-ideal internalization and body dissatisfaction through the general appearance comparison for late adolescent boys and girls separately. The findings of the present study may facilitate identifying individuals who are vulnerable to body dissatisfaction earlier, before more serious eating problems occur.

The following hypotheses were formulated:

1. Gender differences in thin-ideal internalisation, muscular-ideal internalization, general appearance comparison and body dissatisfaction exist with higher levels among adolescent girls with the exception of muscular-ideal internalization.
2. There is an indirect association between thin-ideal internalisation and body dissatisfaction through the general appearance comparison among late adolescent girls and boys.
3. There is an indirect association between muscular-ideal internalization and body dissatisfaction through the general appearance comparison among late adolescent girls and boys.

## Method

### Sample and Procedure

A convenience sampling method was used for data collection. Data were collected by a team of trained researchers and their assistants at four grammar schools in the east of Slovakia in February 2020. The respondents filled in questionnaires during regular school lessons on a voluntary and confidential basis without the presence of a teacher. The study obtained local university ethic committee approval.

The sample consisted of 154 adolescents aged between 17 and 20 years (mean age 18.2 years, *SD* = 0.73; 56.5% girls). Most of them (77.9%) were in the fourth grade, 13% in the third grade and 9.1% in the second. Most of the respondents (92.9%) lived with their parents during the school year, others lived in a dormitory (3.9%), with their grandparents (2.6%) or other places (0.6%). Most of their parents worked full time or part time (both parents working – 82.5%, only mother working – 6.5%, only father working – 9.7%). Regarding their financial situation, 46.1% evaluated their financial situation the same, 40.9% a bit better, 6.5% a bit worse, 5.8% much better and 0.6% much worse in comparison to other families.

### Measures

All the measures used in the study were back translated to Slovak by a native speaker. Any inconsistencies in any particular items were then discussed by two experts in health psychology and the items were subsequently adjusted. A mean score of each measure was calculated and included in the analyses. Table 1 shows the descriptive statistics and gender differences of all the explored variables while Table 2 presents the internal consistencies of all the subscales which were found to be acceptable.

**Table 1:** Descriptive Statistics and Gender Differences in the Explored Variables

	*N*		*M*		*SD*		*Min – Max*					
Variable	Boys	Girls	Boys	Girls	Boys	Girls	Boys	Girls	*t*	*df*	*p*	η^2^
Body dissatisfaction	67	87	2.12	2.11	0.73	0.71	1–4.2	1–4.76	0.094	152	.93	< .01
Body comparison	67	87	2.31	2.08	0.74	0.69	1–4.04	1–4.48	1.992	152	.048*	.03
Thin-ideal internalization	67	87	2.57	2.76	0.93	0.84	1–4.4	1–5	-1.366	152	.17	.01
Muscular-ideal internalization	67	87	3.34	2.63	0.96	0.97	1.4–5	1–5	4.517	152	< .001**^a^	.12

^a^The results are significant even after the Bonferroni adjustment (after the application of the Bonferroni adjustment, the threshold for testing the individual hypotheses was set to .013).

*p < .05. **p < .001. One-tailed *p* values are reported.

**Table 2:** Correlations and Cronbach’s Alpha Values

Variable	1	2	3	4	α*_boys_*
1. Body dissatisfaction	*—*	.49**^a^	.18	-.08	.93
2. Appearance comparison	.65**^a^	*—*	.14	.29*	.93
3. Thin-ideal Internalization	.30**^a^	.36**^a^	*—*	.15	.80
4. Muscular-ideal Internalization	.18	.24*	.45**^a^	*—*	.87
α*_girls_*	.93	.93	.93	.93	—

*Note.* Correlations for girls (*N* = 87) are presented below the diagonal; correlations for boys (*N* = 67) are presented above the diagonal.

^a^The results are significant even after the Bonferroni adjustment (after the application of the Bonferroni adjustment, the threshold for testing the individual hypotheses was set to .013).

*p < .05. **p < .001.

The Body Comparison Scale (Thompson et al., 1999) is a 25-item measure which assesses a respondent's tendency to compare specific body parts and body attributes (e.g., height, weight, body shape, waist, hips, thighs, stomach, face, body build, shoulders) to those of others of the same sex. Respondents indicate how often they engage in each comparison using a 5-point Likert scale from 1 (never) to 5 (always). Higher scores reflect more frequent engagement in general social appearance comparisons. This cognitive comparison process has been examined by the same scale in a later publication which supported its acceptability and usefulness (Fisher et al., 2002).

To measure body dissatisfaction, items of the previous scale (Body comparison scale, Thompson’s et al., 1999) were adapted to indicate participant’s degree of satisfaction with 25 body parts and body attributes (e.g., height, weight, body shape, waist, hips, thighs, stomach, face, body build, shoulders) on a 5-point response scale ranging from 1 (very satisfied) to 5 (very dissatisfied). Higher scores indicate greater body dissatisfaction.

Thin-ideal Internalization and Muscular-Ideal Internalization were measured by two subscales in the Sociocultural Attitudes Towards Appearance Questionnaire-4 (SATAQ-4) (Schaefer et al., 2015): Internalization: Thin/Low Body Fat (sample item: “I think a lot about looking thin.”) and Internalization: Muscularity/Athletic (sample item: “It is important for me to look athletic.”). These subscales assess the endorsement and acceptance of media messages which promote unrealistic ideals of thinness and muscularity (muscular or toned physique) and striving towards these ideals. Each scale consists of 5 items, rated from 1 (definitely disagree) to 5 (definitely agree). The total score is created by summing all items. Higher scores reflect greater internalization of the ideals conveyed by the media. The scale has also been used multiculturally and publications yielded acceptable psychometric characteristics (e.g., Cihan et al., 2016; Neves et al., 2020; Zevallos-Delzo et al., 2020).

### Analyses

In order to describe the sample and examine the gender differences, a descriptive analysis, Pearson correlations and t-tests were performed in SPSS 20. A Bonferroni adjustment was applied to the individual alpha levels to deal with multiple hypotheses testing (4 one-tailed tests addressing gender differences). Since the overall alpha level was set to .05, the individual relationships were considered statistically significant if the corresponding one-tailed *p*-values were lower than .05/4 = .013. The strength of gender differences was also interpreted according to the effect size guidelines proposed by Cohen (1988, as cited in Pallant, 2007).

**Figure 1:**
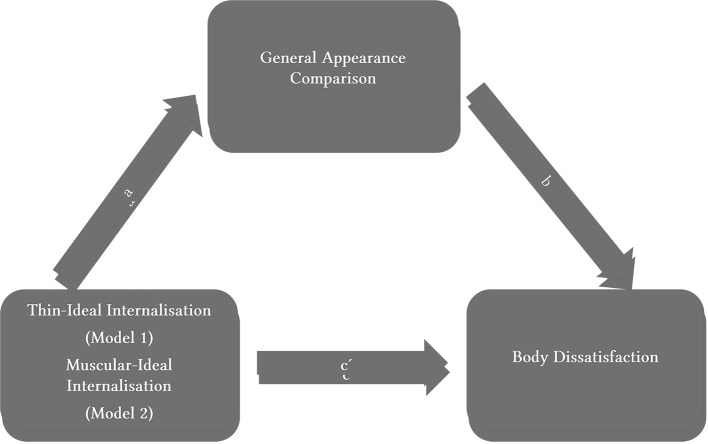
Hypothetical Model of the Effect of Ideal Body Internalisation [Thin-Ideal (Model 1), Muscular-Ideal (Model 2)] on Body Dissatisfaction Through the General Appearance Comparison

Two hierarchical regression models were conducted to analyse the indirect effect of body ideal internalization: Thin-ideal (Model 1), Muscular-ideal (Model 2), on body dissatisfaction through the general comparison of appearance (Figure 1) for boys and girls separately. The analyses were conducted using PROCESS Macro in SPSS which is a conditional process modelling program that utilizes an ordinary least squares regression-based path analysis to test for direct and indirect effects (Hayes, 2013).

## Results

### Preliminary Analysis

Table 1 shows the descriptive statistics and gender differences of all the explored variables. The boys scored higher on the body comparison (*p* = .048, η^2^ = .03) and muscular-ideal internalization (*p* < .001, η^2^ = .12; significant even after the Bonferroni adjustment). However, the magnitude of differences in the means was small to moderate according to the effect size. Other gender differences were not significant. Table 2 presents the correlations between the variables as well as the internal consistencies of all the subscales which were found to be acceptable. The appearance comparison was found to be significantly strongly positively associated with body dissatisfaction (even after the Bonferroni adjustment, *r* = .49, *p* < .001) and weakly positively associated with muscular-ideal internalization (*r* = .29, *p* < .05) among boys. Body dissatisfaction was significantly strongly positively correlated with the appearance comparison (*r* = .65, *p* < .001) and moderately with thin-ideal internalization (*r* = .3, *p* < .001) among girls. Thin-ideal internalization was also moderately positively associated with the appearance comparison (*r* = .36, *p* < .001) among girls. Muscular-ideal internalisation was moderately positively associated with thin ideal internalization (*r* = .45; *p* < .001) as well as with the appearance comparison (*r* = .24, *p* = .05, small correlation, not significant after Bonferroni adjustment) among girls.

### Exploration of the Complex Relationships in the Models

The partial correlations among the girls were significant with a moderate to large effect regarding thin-ideal internalization and other variables. On the contrary, moderate to strong correlations were found regarding muscular-ideal internalization among boys. Although the correlations between muscular-ideal internalization and body dissatisfaction were not significant for either boys or girls, the correlation between muscular-ideal internalization and appearance comparison was small but significant (Table 2). This creates a basis for testing the mediation effect of appearance comparison in the relationships between both thin and muscular-ideal internalization and body dissatisfaction among girls and muscular-ideal internalization among boys.

The first regression model tested whether social comparison acted as a mediator between thin-ideal internalisation and body dissatisfaction separately for boys and girls. Among the girls, both the total effect model, *R*^2^ = .09, *F*(1, 85) = 8.48, *p* = .01, and the direct effect model with appearance comparison as the mediator, *R^2^* = .43, *F*(2, 84) = 31.22, *p* < .001, were significant. Greater thin-ideal internalization predicted a greater level of social comparison (Path A) and greater social comparison predicted greater body dissatisfaction (Path B). In terms of testing the indirect effect, thin-ideal internalization was predictive of greater body dissatisfaction indirectly through social comparison, indirect effect = .19; *SE* = .10; 95% CI [.05, .37].

Among the boys, the total effect model was not significant, *R*^2^ = .03, *F*(1, 65) = 2.23, *p* = .14, while the direct effect model with the appearance comparison as the mediator was significant, *R^2^* = .25, *F*(2, 64) = 10.81, *p* < .001. There was no significant association found between thin-ideal internalization and social comparison (Path A) among the boys, nor was there a significant indirect effect between thin-ideal internalization and body dissatisfaction through social comparison, indirect effect = .05; *SE* = .06; 95% CI [-.04, .19]. However, greater social comparison was associated with greater body dissatisfaction (Path B).

The second regression model tested whether social comparison acted as a mediator between muscular-ideal internalisation and body dissatisfaction for boys and girls separately. Among the girls, the total effect model was not significant, *R^2^* = .03, *F*(1, 85) = 8.48, *p* = .09, although the direct effect model with the appearance comparison as the mediator was significant, *R^2^* = .42, *F*(2, 84) = 30.67, *p* < .001. Greater muscular-ideal internalization predicted a greater level of social comparison (Path A) and greater social comparison predicted greater body dissatisfaction (Path B). In terms of testing the indirect effect, muscular-ideal internalization was predictive of greater body dissatisfaction indirectly through social comparison, indirect effect = .11; *SE* = .07; 95% CI [.007, .26].

Among the boys, the total effect model was not found to be significant, *R^2^* = .007, *F*(1, 65) = .45, *p* = .5, although the direct effect model with the appearance comparison as the mediator was significant, *R^2^* = .29, *F*(2, 64) = 13.34, *p* < .001. Greater muscular-ideal internalization predicted a greater level of social comparison (Path A) and greater social comparison predicted greater body dissatisfaction (Path B). In terms of testing the indirect effect, muscular-ideal internalization was predictive of greater body dissatisfaction indirectly through social comparison, indirect effect = .12; *SE* = .05; 95% CI [.03, .24]. On the contrary, the direct effect between body dissatisfaction and muscular-ideal internalization was significant and negative (Path C´) after the mediator was included in the model.

Regression weights for Paths A, B, C, and C´ are presented in Table 3 for each of the regression models.

**Table 3:** Regression Results for the Indirect Effects

Model	Path	*b*	*SE*	*t*	*p*	95% CI
Model 1 (girls)	a	.3	.08	3.58	<.001	[.13, .47]
	b	.64	.09	7.01	<.001	[.46, .82]
	c	.26	.09	2.91	.01	[.08, .43]
	c´	.06	.08	.86	.39	[-.09, .21]
Model 1 (boys)	a	.12	.1	1.17	.24	[-.08, .31]
	b	.47	0.11	4.33	<.001	[.25, .68]
	c	.14	.1	1.49	.14	[-.05, .34]
	c´	.09	.09	1.04	.3	[-.08, .26]
Model 2 (girls)	a	.17	.08	2.3	<.02	[.02, .32]
	b	.66	.09	7.52	<.001	[.49, .83]
	c	.13	.08	1.7	.09	[-.02, 29]
	c´	.02	.06	.31	.76	[-.11, .15]
Model 2 (boys)	a	.22	.09	2.42	.02	[.04, .40]
	b	.55	.11	5.10	<.001	[.34, .77]
	c	-.06	.09	-.67	.5	[-.25, .13]
	c´	-.19	.08	-2.22	.03	[-.35, -.02]

*Note*. Model 1—The indirect effect of thin-ideal internalization on body dissatisfaction through the general comparison of appearance. Model 2. The indirect effect of muscular-ideal internalization on the body dissatisfaction through the general comparison of appearance.

## Discussion

The main aim of the current study was to explain the association between thin-ideal internalisation, muscular-ideal internalization and body dissatisfaction through the general appearance comparison separately for adolescent boys and girls. Moreover, gender differences in these concepts were examined as well. The presented findings partially supported the first hypothesis, with regards to muscular-ideal internalization. Other gender differences were not found. While the second hypothesis considering the indirect association between thin-ideal internalization and body dissatisfaction was partially supported (only among girls) the third hypothesis regarding muscular-ideal internalization was fully supported by the results. The current findings support and contribute to previous research findings, particularly with regard to focusing on both genders and late adolescence and are discussed in detail in the following text.

The boys in the current study reported higher levels of appearance comparison than the girls. Even though the difference was small, this result is uncommon as it tends to be women who score higher on body comparison scales (e.g., Ko et al., 2019; Schaefer & Blodgett Salafia, 2014; Watson & Murnen, 2019). It seems that social context and cultural glorification play a distinct role for men and women at different ages. While girls overestimate the importance of social appearance from an early age, boys start to find it relevant in late adolescence (Carlson Jones, 2004). Indeed, the current study has indicated that they find it even more relevant than girls at this age. Appearance comparison could be more an intense process for boys at this age, while girls have already managed this issue or have dealt with it longer.

The boys in the current study also scored slightly higher on muscular-ideal internalization which is in line with expectations as well as with previous research findings (Schaefer & Blodgett Salafia, 2014). This also reflects the typical body ideal for men in western culture (Tylka, 2011). However, it was surprising to find that there were no significant gender differences in body dissatisfaction and thin-ideal internalization (in contrast with Knauss et al., 2007; Paulisova et al., 2021; Schaefer & Blodgett Salafia, 2014). These inconsistent findings regarding body dissatisfaction could reflect the nature of the scale used for body dissatisfaction in the current research. While specific body parts and body attributes were assessed here, other scales usually tend to evaluate global body dissatisfaction. Nevertheless, the current findings emphasize the need for men to be included more in these types of studies (Ko et al., 2019).

The indirect effect between thin-ideal internalization and body dissatisfaction through the appearance comparison among boys has not been supported by the current results. There was no significant association found between thin-ideal internalization and social comparison among boys, nor was there a total effect of thin-ideal internalization on body dissatisfaction. The thin-body ideal seems to be irrelevant in the body dissatisfaction of boys which corresponds with their muscular-body ideal found in recent research (Flament et al., 2012; Knauss et al., 2007). General appearance internalization has previously been found to be insignificant in explaining body dissatisfaction among undergraduate men (Hricová & Orosová, 2017). However, greater social comparison was found to be associated with greater body dissatisfaction among boys in the current study. Similarly, the appearance comparison has previously been positively correlated with body dissatisfaction among early and middle adolescent boys (Carlson Jones, 2004; Ho et al., 2016), young adult men (Modica, 2020) and negatively with body esteem tested on a mixed gender sample of young adults (Ko et al., 2019). Social comparison online has been found to be significantly associated with body image dissatisfaction of middle adolescents for both genders (Ho et al., 2016).

On the other hand, the indirect effect of muscular-ideal internalization on the body dissatisfaction of boys through the appearance comparison has been found to be significant in the current study. Moreover, the direct effect between muscular-ideal internalization and body dissatisfaction after the mediator was included in the model was significant among boys as well, although the direct effect was negative. This means that the more boys internalize the muscular body ideal, the less they feel dissatisfied with their body. On the contrary, previous findings have shown that adolescent boys with greater muscular-body ideal internalization were more likely to express greater body dissatisfaction (Carlson Jones, 2004; Knauss et al., 2007) and lower body esteem (Flament et al., 2012). Tylka (2011) found that internalization of the mesomorphic ideal was positively associated with muscularity dissatisfaction and body fat dissatisfaction among undergraduate men. Besides the study design, it is possible to explain this inconsistency by the muscular-ideal internalization subscale used in the current study; it includes cognitive-internalization aspects as well as items that describe behavioural aspects—an effort to gain a muscular and athletic figure. The more boys think about being muscular and the more effort they make to look muscular, the less dissatisfied they are with their body. Regarding the indirect effect, a greater acceptance of media messages about muscular figures and a greater effort to fulfil them might lead to more appearance comparison with their peers to see how successful they are at this. According to the current results, general comparison seemed to enhance body dissatisfaction. These results suggesting a negative impact of the general appearance comparison are consistent with the previously found association between general appearance comparison and body dissatisfaction (Tiggemann & McGill, 2004). However, the peer appearance context was not found to be relevant in a more complex model explaining body dissatisfaction among early and middle adolescent boys, explained by the increasing amounts of time boys spend alone during adolescence (Carlson Jones, 2004). Contrary to this, the current research addressing late adolescent boys brings findings which support the importance of the social environment in body dissatisfaction development. The current results thus reflect the developmental delay among boys regarding appearance comparison. These tend to arise later in comparison to girls who are involved in the appearance culture from an early age (Carlson Jones, 2004). In the context of the Tripartite Influence Model of Body Image and Eating Disturbance (Thompson et al., 1999), the current results support the significant role of social appearance comparison and muscular-ideal internalization in the development of body dissatisfaction among adolescent boys, consistent with previous testing of the model on undergraduate men (Carvalho & Ferreira, 2020; Tylka, 2011). Moreover, the current findings also indicate that social comparison could mediate the association between muscular-ideal internalization and body dissatisfaction among boys. This has also been outlined in the latest refinement of the Tripartite Influence Model among undergraduate men (Schaefer et al., 2021).

Among the girls, the appearance comparison was associated with body dissatisfaction and both muscular-ideal and thin-ideal internalization. Body dissatisfaction was correlated with thin-ideal internalization among the girls but not with muscular-ideal internalization. An exploration of the more complex associations has shown an indirect effect between thin-ideal internalization as well as muscular-ideal internalization and body dissatisfaction through the appearance comparison among girls. Greater ideal internalization predicted a greater level of social comparison and greater social comparison predicted greater body dissatisfaction. The general body comparison has been found to play a role of the strongest mediator between thin-ideal internalization and body dissatisfaction among undergraduate women, pre-adolescent and middle adolescent girls (Blowers et al., 2003; Fitzsimmons-Craft et al., 2016), consistent with the current findings. However, testing university-aged women failed to produce that same effect (Fitzsimmons-Craft et al., 2012). For adolescent girls, thinness and physical beauty are socially desirable attributes (Giles, 2003). On the other hand, the ideal women’s body conveyed by the media also focuses on muscularity, athleticism and exercise (Tylka, 2011). However, the relationship between the thin-ideal internalization and body dissatisfaction seem to be stronger than the association between muscular-ideal internalization and body dissatisfaction, which could be explained by the easier attainability of the muscular body-ideal (Paterna et al., 2021). This could also explain the absence of the significant association between the muscular-ideal internalization and body dissatisfaction among girls in the current study. While athletic-ideal internalization has not been directly associated with body dissatisfaction or the association was of a small effect in previous studies, it has been found to predict unhealthy weight control behaviour among young adult women (Bell et al., 2016). Similarly, the current results have also not found direct associations although they have revealed an indirect relationship between muscular-ideal internalization and body dissatisfaction. Consistently with the current findings, positive associations between upward and downward appearance comparison, body dissatisfaction and general ideal internalization among undergraduate women has previously been found (Vartanian & Dey, 2013). Social comparison and generally internalized appearance ideals have predicted prospective changes in body dissatisfaction among adolescent girls in longitudinal study designs as well (Carlson Jones, 2004; Rodgers et al., 2015). Furthermore, physical appearance comparison has been shown to be strongly associated with disordered eating symptoms (Alcaraz-Ibáñez et al., 2020). The current results support the importance of appearance comparison and both types of appearance ideals internalization among adolescent girls in the development of body dissatisfaction.

To sum up, the general appearance comparison works as an important explanatory mechanism of the relationship between both thin-ideal internalization and muscular-ideal internalization and body dissatisfaction. The extent to which individuals compared their physical appearance to others mediated the relationship between their thin/muscular-ideal internalization and body dissatisfaction. The appearance comparison seems to be the risk factor which enhances body dissatisfaction among both boys and girls in late adolescence.

### Limitations

It is also important to acknowledge the limitations of the current study. One limitation is the cross-sectional design used to test the mediation model. Longitudinal research would be of value in order to test the obtained results and address the causality of the relationships. However, the rationale of temporal ordering of the examined variables is clearly stated in the hypothetical model. Thus, examining the mediation hypotheses with cross-sectional data is reasonable (Fairchild & McDaniel, 2017). Other inaccuracies could have been caused by the self-reporting nature of the data which can be biased by social desirability, especially in the topic of “social ideals”. Another limitation is using the general appearance comparison scale, even though there is a tendency of upward comparisons regarding physical appearance and there is evidence of a negative impact of the general appearance comparison on body satisfaction. In future research, distinguishing between the upward and downward comparison scales could be used to bring more specific findings. Finally, while the methods of the Body Comparison Scale and SATAQ-4 are standardized and widely used abroad, the scale used to measure body dissatisfaction needs further exploration of the internal structure, validity and reliability. However, scales using the assessment of one’s own body parts in order to catch the extent and focus of body dissatisfaction are often used (Ricciardelli & Yager, 2016). Further validation of all used measures in the population of Slovak adolescents might improve their psychometric qualities.

### Implications and Conclusions

A direct effect between thin-ideal internalisation and body dissatisfaction was not significant among either the girls or the boys. An indirect effect through the general appearance comparison was found among the girls where greater thin-ideal internalization predicted a greater level of social comparison and greater social comparison predicted greater body dissatisfaction. Among the boys, greater social comparison was only associated with greater body dissatisfaction.

An indirect association between muscular-ideal internalization and body dissatisfaction through the general appearance comparison was found among both the boys and the girls. The direct effect was not significant for the girls but was significant and negative for the boys. According to the current results however, the general comparison seemed to enhance body dissatisfaction for both genders.

This study has provided new findings regarding the functionality of a more complex model of body dissatisfaction among late adolescent boys. The cultural context seems to be important at this age. The results have contributed to the current state of research on this issue regarding the developmental period of late adolescence. The appearance comparison seems to be the risk factor which enhances body dissatisfaction among both boys and girls in late adolescence. Finally, the current results have supported the relevance of both types of societal ideal internalization (thin-ideal and muscular-ideal) in the body dissatisfaction of girls.

The internalization of body ideals among men and women contributes to body dissatisfaction, unhealthy weight control strategies and disordered eating (Bell et al., 2016; Brown et al., 2017). As is already known, men are less likely to admit a problem and seek treatment (Brown et al., 2017), partially due to social stigma. The current findings may facilitate identifying individuals who are vulnerable to body dissatisfaction earlier, before more serious eating problems occur. Body dissatisfaction prevention programs might benefit from the findings by focusing on more realistic perceptions of physical appearance in comparison to those conveyed by the media. It has been found that critical processing of unrealistic perfect body ideals helps both genders (Watson & Murnen, 2019). Similarly, such programs might emphasize individual norms of performance and appearance evaluation in order to prevent or interrupt the negative effects of social comparison.

Future research could take advantage of a longitudinal study design to verify the mediational effect of the appearance comparison. In addition, the differentiation of the appearance comparisons into upward and downward comparisons might reveal more specific processes. The current findings in addition to the previous ones highlight the importance of exploring both genders in such a topic including both the thin and muscular body ideals.
